# How to Repair Non-Atheromatous Carotid Lesions

**Published:** 2020-02-20

**Authors:** NM Bouayed, LA Bouziane

**Affiliations:** Vascular Surgery Department University Hospital Hai Sabah, Oran, Algeria

**Keywords:** carotid lesions, fibromuscular dysplasia, carotid aneurysms, Behcet’s disease, carotid stent

## Abstract

Non-atheromatous surgical lesions are estimated to represent at most 10% of all carotid procedures, most of which involve atheromatous lesions. Isolated tortuosity of the carotid vessels is sometimes treated surgically. The pathologies most frequently studied are extra-cranial carotid aneurysms, dissections, and fibromuscular dysplasia. Behcet’s disease only rarely affects the carotid trunk, but in view of its prevalence in our country of Algeria a short section will be devoted to it. A series of 57 patients treated for non-atheromatous carotid lesions is presented article. These cases were treated using both endovascular and conventional surgical techniques. A review of the literature shows that endovascular treatment is now replacing conventional surgery for most indications except carotid paraganglioma.

## I. INTRODUCTION

Atheromatous stenosis represents the majority of carotid lesions. Nevertheless, approximately 10% of surgical procedures on the carotid are performed for other causes [1]: kinkings, radiation-induced stenosis, restenosis and false aneurysms (FA) after endarterectomy, FA after closed trauma, carotid paraganglioma, dissections, fibromuscular dysplasia (FMD), extra-cranial arterial aneurysms (ECAA) and carotid complications of Behcet’s disease (BD). Occurrence of a stroke is the major risk in the evolution of these lesions. Improvements in equipment and techniques have led to endovascular treatment being used more and more frequently for these carotid lesions.

We treated 57 patients at our department for non-atheromatous carotid lesions. Among them, 6 were FA related to BD which is relatively frequent in our geographical region. Arterial complications during treatment and follow-up represent 7% of all our vascular Behcet cases. In 80% of these cases they took the form of FA with a high risk of recurrence after treatment. Localization in the carotid is relatively rare compared with other aortic, pulmonary and peripheral sites. Over the last 14 years we have operated 30 cases of arterial complications in BD, including 4 carotid cases (13.3%). The occlusive, or very rarely aneurismal, lesions associated with Takayasu’s disease that are located preferentially in the supra-aortic trunks are excluded from our series, as are open traumatic carotid lesions.

The aim of this work is to present, through the study of our series and a review of the literature, the current state-of-the-art in the treatment of non-atheromatous carotid lesions.

## II. PERSONAL SERIES

57 cases of non-atheromatous carotid lesions were treated in our department. The table below summarizes the data in the patient files (table 1).

For FA linked to BD, sutures are systematically reinforced by prosthetic pledgets, and anastomoses by bands of prosthetic tissue. Patients with BD receive mandatory dual therapy: steroid and immunosuppressive treatment.

## III. DISCUSSION

Isolated kinking of the carotids is rarely a surgical indication.

The 3 cases in our series of restenosis after endarterectomy which remained asymptomatic were simply kept under observation during follow-up. However, the others, and above all the symptomatic cases, are now treated by dilation and stenting^2^. FA caused by disinsertion of an angioplasty patch are treated with good results by implantation of a covered stent 3. Endovascular treatment by angioplasty and stenting has been extensively validated for the treatment of radiation-induced stenosis^4^. Carotid paraganglioma is treated surgically. In some cases pre-operative embolization makes it possible to reduce the size of some large tumours and reduce intra-operative bleeding.

Acute dissections are generally treated by anticoagulants however Ohta reported a series of 44 cases of carotid dissection treated by stenting with a favourable outcome in 83.7% of cases 5. We believe however that only the aneurismal complications of carotid dissection can benefit from surgery or an endovascular procedure.

FMD is a rare, segmental, non-atherosclerotic and non-inflammatory idiopathic arteriopathy affecting medium-sized vessels in women aged 30 to 50 6. Lesions of the internal carotid are rare and generally asymptomatic 7. However, this type of dysplasia may be complicated by dissections, dilation due to aneurysm and transient or permanent stroke 6,7.

The prevalence of carotid FMD, as assessed by angiography, is only 0.5 – 0.7% 8.

Three types have been identified: type 1 giving rise to the typical “pearl necklace” appearance; type 2, less frequent, giving rise to an image of a short focal stenosis (present in the only patient in our series who underwent surgery) or a long tubular lesion; and type 3 characterized by a dysplastic aneurysm 6.

Once the indication has been decided, carotid FMD is increasingly being treated by angioplasty and stenting with good long-terms results 8.

We have also treated 12 cases of extra-cranial carotid aneurysms (ECAAs) of which 5 were spontaneous and 7 post-traumatic.

ECAAs are rare. They represent between 0.4% and 4% of all peripheral aneurysms and only 0.1% to 2% of all carotid surgical procedures 1.

For ECAAs (true or false aneurysms), the surgical procedures involve resection with, according to the situation, a direct anastomosis, possible when there is elongation of the carotid, or interposition of a vein graft when the aneurysm is located at the level of the internal carotid, or interposition of a prosthesis when it is located at the level of the common carotid artery. Resection of saccular aneurysms is often followed by an end-to-end anastomosis; sometimes, when the neck is narrow, a lateral suture is possible. ECAAs, when they are situated high up near the base of the skull, can now be treated using endovascular techniques. Many authors have reported series of ECAAs treated by covered stents with good results 9. Li published a compilation of several series totaling 224 cases where an ECAA was treated by implantation of covered stents. The initial success rate was 92.8%, with a 1.8% rate of established ischemic stroke and a mortality rate of 4.1%. The medium-term patency rate was 93.2% 10. One of our patients was treated using an endovascular approach. The ECAAs were accessible through a cervical approach in patients with low operative risk. Extra-cranial carotid FA secondary to BD are extremely rare. According to Berard only 32 cases have been reported in the literature [11]. Our experience is that the most effective surgical technique is resection with a lateral or end-to-end suture of the native artery reinforced by synthetic tissue pledgets, or with interposition of a PTFE prosthesis, always reinforced by a band of prosthetic tissue surrounding the artery at the level of the anastomosis. In all cases an antithrombotic is prescribed, combined with long-term dual therapy: prednisone at a dose of 1mg/Kg/day quickly reducing to a maintenance dose of 10 to 15mg/day and an immunosuppressive drug. We use azathioprine at a dose of 2.5mg /kg/day. This dual therapy is necessary to prevent local recurrence and the recurrence of other FA in other territories. A vein graft as an arterial substitute is not adequate because venous material itself can suffer degenerative modifications due to BD, leading to occlusion or FA in the graft 11. Endovascular treatment of FA in a context of BD has been reported but the very limited number of cases does not allow any conclusion to be drawn from the results 12. One of our BD patients was treated by implantation of a covered carotid stent with a good result but 50% of the stents placed in other peripheral sites became thrombotic within 18 months. Finally, a simple reinforced ligation of the internal carotid is used by some authors when the lesions are in a high position, not susceptible to easy repair and provided there is good collateral circulation as evidenced by a sufficient residual pressure 13.

## IV. CONCLUSION

Non-atheromatous carotid lesions are infrequent. They should be treated when they are symptomatic: compressive signs and focal neurological accidents. However, an aneurysm, even asymptomatic, should be operated on if imaging studies reveal the presence of thrombus on its walls. Numerous surgical procedures are available and are adapted to the morphology and anatomy of specific lesions. However, it must be acknowledged that endovascular techniques are now tending to replace conventional surgery with results that are continually improving.

## Figures and Tables

**Table 1:** Summary of Lesions, Clinical Signs, Treatments and Outcomes for our Patients

Lesions	Number of cases	Clinical signs	Treatment	Outcome (average follow-up 6 yrs)
Radiation-induced stenoses	5	4 asymptomatic 1 hemiparesis of an upper limb	Medical Endarteriectomy and prosthetic patch	3 Stable and 1 death due to a cancer Favourable
Restenoses	8	7 Asymptomatic 1 restenosis and FA	3 Medical, 4 Stenting 1 Covered Stent	7 favourable Favourable
Carotid paraganglioma	21	Compressive cervical mass	9 resections + interposition of a prosthesis 1 internal carotid ligation 12 complete resection with no carotid procedures	3 lesions of peripheral nerves regressing in a few weeks 2 regressive stroke Good evolution otherwise
Fibromuscular dysplasia(FMD)	5	4 transient ischemic accident 1 total hemiplegia	1 Resection and vein graft and 4 medical treatment	4 Favouroble 1 Sequelae of stroke
Spontaneous aneurysms out BD	5	5 compressive cervical masses	2 resections + interposition of a prosthesis 3 resection and end-to-end suture	Favourable
Post-traumatic aneurysms	7	5 compressive cervical masses 1 carotido-jugular aneurysm 1 cervical mass with paresis of an upper limb	3 resection and end to end anastomosis 4 resection + interposition of a graft	Favourable in 6 cases 1 death following massive stroke
FA secondary to BD	6	6 cervical masses with signs of compression in 4 cases, signs of suffocation in 1 case (emergency surgery and one case of minor sequelae of a stroke	1 lateral suture with patch 1 end-to-end anastomosis 3 interposition of a Dacron prosthesis 1 Covered stent	1 death following a respiratory complication 5 favourable without recurrence
